# A Description and Comparison of Cardiorespiratory Fitness Measures in Relation to Pitching Performance Among Professional Baseball Pitchers

**DOI:** 10.3390/sports4010014

**Published:** 2016-02-25

**Authors:** Javair S. Gillett, J. Jay Dawes, Frank J. Spaniol, Matthew R. Rhea, Joe P. Rogowski, Mitchel A. Magrini, Roberto Simao, Derek J. Bunker

**Affiliations:** 1 Athletic Performance, Houston Rockets, Houston, TX 77002, USA; jgillett@rocketball.com; 2 University of Colorado, Colorado Springs, CO 80918, USA; jdawes@uccs.edu; 3 Texas A&M University-Corpus Christi, Corpus Christi, TX 78412, USA; 4 A.T. Still University, Kirksville, MO 63501, USA; mrhea@atsu.edu (M.R.R.); Bunkjames7@hotmail.com (D.J.B.); 5 Athletic Heart Research Institute, Orlando, FL, 32801, USA; jrogowski@athletic-heart.com; 6 Oklahoma State University, Stillwater, OK 74074, USA; mmagrini@uccs.edu; 7 Federal University of Rio de Janeiro, Rio de Janeiro 21941-901, Brazil; rsimaoj@terra.com.br

**Keywords:** conditioning, endurance, pitching, performance, VO_2 max_

## Abstract

The purpose of this study is to provide descriptive and comparative information regarding the cardiorespiratory fitness of professional baseball pitchers. Twenty-four (*n* = 24) major league (ML) baseball pitchers (starters *n* = 14; relievers *n* = 10) over seven seasons (2007–2013) were evaluated. A modified Bruce protocol and the CardioCoach™ CO_2_ metabolic analyzer were used to estimate VO_2 max_ and anaerobic threshold (AT) at the beginning of each season. Performance data from each season was utilized to draw inference about pitching performance. One-way Analysis of Variance (ANOVA) was used to compare Starting (S) and Relief (R) pitchers above/below the group mean for VO_2 max_ and AT. Pearson product moment correlations were also used to examine relationships between cardiorespiratory fitness and performance. Significant differences in performance were discovered between S pitchers above/below the overall group mean for VO_2 max_. (*p* ≤ 0.05) and for AT in Walks plus Hits per Inning Pitched (WHIP) (*p* ≤ 0.05) and Earned Run Average (ERA) (*p* ≤ 0.05). Significant relationships between VO_2 max_ and Walks per 9 Innings (BB/9) (*p* ≤ 0.05), Home Runs per 9 innings (HR/9) (*p* ≤ 0.05), Wins (W) (*p* ≤ 0.05), Fielding Independent Pitching (FIP) (*p* ≤ 0.01), Strikeouts (K) (*p* ≤ 0.01), Hits per 9 innings (H/9) (*p* ≤ 0.01), Strikeouts per 9 innings (K/9) (*p* ≤ 0.01), ERA (*p* ≤ 0.01), and WHIP (*p* ≤ 0.01). Low, but significant, correlations were discovered between AT and WHIP (*p* ≤ 0.05) and ERA (≤0.05). CONCLUSION: Higher aerobic capacity appears to be more influential for S than R pitchers. Strength and conditioning practitioners should ensure that pitchers, especially S pitchers at the ML level, perform sufficient and appropriate endurance training to support pitching performance.

## 1. Introduction

Baseball is a sport that requires short, explosive bursts of intense effort. While the duration of each play is relatively short, a typical professional baseball game takes approximately three hours to complete [1]. During a game, only two of the nine players on the field are involved in every single play, the pitcher and catcher. While catchers perform many of their skills at submaximal intensities, pitchers are expected to deliver every pitch at maximum, or near maximum effort [2]. A starting pitcher typically delivers 80–100 pitches or more per game, whereas a relief pitcher is required to throw significantly fewer pitches per performance (40 or less). Whereas a starting pitcher is typically allowed four days between performances, a reliever might be required to throw on consecutive days.

Pitching relies heavily on the ATP-PCr system during the delivery of the pitch, followed by brief bouts (approximately 20–30 s) of aerobic recovery between pitches [3]. Subsequently, pitching appears to place a relatively low demand on the aerobic energy system [4]. In fact, Potteiger *et al*. [4] found that mean oxygen consumption ranged between 14.8–20.6 mL·kg ^−1^ min ^−1^ for pitchers pitching in a simulated game. The researchers noted that this would correspond to a continuous exercise intensity at approximately 45% of the participants mean VO_2 max_. However, since this study was performed in a laboratory setting where the pitchers did not face game competition, one may speculate that the aerobic demands may differ during actual competition.

Stockholm and Morris [5] conducted a study in which a freshman collegiate baseball pitcher’s heart rate (HR) was monitored and recorded during competition (3 h and 10 min, 9 inning game) via the use of telemetry. It was discovered that the mean heart rate during the performance was approximately 87% of the player’s age-predicted heart rate max (HR _max_), with peak HR reaching 95% of the player’s age-predicted HR max. This is significantly greater than HRs achieved during a laboratory study [4] and during bullpen practice sessions prior to an intra-squad game [6]. This suggests that arousal and anxiety levels may impact the physiological demands of pitching. Furthermore, being that the bulk of competitions in professional baseball occur during the summer months, greater cardiorespiratory fitness may help players better accommodate the physiological challenges and delay the onset of fatigue when playing in hot/humid environments [5,6,7,8,9,10,11,12,13].

Very few studies have investigated the relationship between aerobic fitness and pitching performance [4]. This may in part be due to the observations that baseball is predominantly an anaerobic sport. Ebben *et al.* [14] found that the majority of major league strength coaches do not test anaerobic capacity or aerobic endurance, which may be due to the belief that possessing a high level of aerobic fitness does not appear to limit performance amongst professional or collegiate pitchers. Furthermore, studies that have investigated aerobic fitness and pitching performance have quantified performance in terms of maintenance of ball velocity, rather than the player’s actual game statistics [4]. While one may assume that the maintenance of ball velocity is an inherent predictor of performance, the art of pitching is a multifaceted and complex skill and success should not be limited to a singular variable, particularly at higher playing levels. There are other performance indicators and statistics that are more indicative of individual pitching performance and less dependent on team performance, which should be taken into consideration when seeking to evaluate or predict pitching effectiveness.

Currently, there is very little data available regarding the cardiorespiratory fitness of baseball players, especially at the professional level. Thus, the primary purpose of this investigation was to provide descriptive information regarding the cardiorespiratory fitness profiles of Major League (ML) pitchers, and compare cardiorespiratory fitness levels between starting (S) and relief (R) pitchers. A secondary purpose was to examine the relationship between cardiorespiratory fitness and selected measures of pitching performance.

## 2. Method, Results, Discussion

### 2.1. Methods

#### 2.1.1. Experimental Approach to the Problem

The data used for this study was archival and approved by an Institutional Review Board for research with human subjects prior to data analysis. In order to compare performance between pitchers by position and fitness level and identify the relationships between cardiorespiratory fitness and pitching performance, physiological and performance statistics over seven seasons (2007–2013) for 24 ML baseball pitchers were gathered and analyzed. A correlational analysis was also performed to examine possible relationships between these variables. In addition, statistical analyses were conducted to examine the differences between S and R pitchers.

#### 2.1.2. Subjects

Data for 24 professional pitchers, within the selected ML baseball organization, was utilized in this investigation. Only pitchers remaining within the organization throughout the entire season were included in this analysis. Since some of the subjects in this study completed multiple seasons with the organization, a total of 40 eligible cases (*n* = 27 S; *n* = 13 R) were available for evaluation. For the purpose of analysis, each case (year) was treated separately as both physiological and performance statistics are subsequent to change each season and therefore were evaluated individually. Only pitchers with 50 or more innings pitched per season were used in this study.

#### 2.1.3. Procedures

Physiological testing was performed within the first week of spring training camp each year. All physiological tests were performed after on-field baseball practice on days in which the pitcher did not throw a bullpen session. Pitchers completed a graded, submaximal exercise test on a treadmill (Life Fitness TI, Model 97Ti/Model CLST and Woodway, Model R-DESMO, Franklin Park, IL, USA). The CardioCoach™ CO_2_ metabolic analyzer and software (version 3.04.72, KORR Medical Technologies Inc., Salt Lake City, UT, USA) was used for gas analysis. This device has been found to be a valid method of assessing VO_2_ at submaximal and maximal levels [15]. Subjects performed a modified Bruce protocol and were asked to complete as many stages as possible. The test was terminated when the subject requested to stop or reached volitional fatigue. Utilizing the software provided via CardioCoach™ a linear regression equation was used to predict VO_2peak_. CardioCoach™ software was also used to identify oxygen consumption at anaerobic threshold (AT). In addition to cardiorespiratory fitness, the following anthropometric and physiological data was collected for each of the subjects: height (cm), weight (kg), percent body fat (%BF), and lean body mass (LBM). Body fat was estimated using a six-site skinfold test [16].

Performance data were accessed and gathered immediately after the season via reliable online databases [17,18,19]. These databases are open access sources for pitching statistics. Multiple databases were used for the purpose of comparison to ensure accuracy. The key pitching performance statistics used in this analysis included: Fielding Independent Pitching (FIP), Walks plus Hits per Inning Pitched (WHIP), and Strikeout to Walk Ratio (K/BB). These statistics were chosen because they tend to be the least influenced by uncontrollable variables (*i.e.*, team performance, defense skill, batter skill, situational hitting, stadium environment, *etc.*) and more dependent on the pitcher’s overall pitching performance. Keeping this in mind additional performance statistics analyzed in this study included: Earned Run Average (ERA), Hits per 9 innings (H/9), Homeruns per 9 innings (HR/9), Strikeouts per 9 innings (K/9), and Walks per 9 innings (BB/9), Wins (W), Win/Loss Percentage (W/L%) and Strikeouts (K). Data were entered into a spreadsheet matching performance data with the physiological measures for each player for each year.

#### 2.1.4. Statistical Analyses

A descriptive data analysis was conducted for all pitchers in the sample and by their individual positions (S or R) Comparison between fitness measures among S and R pitchers on selected fitness and performance measures was conducted via an independent samples T-test based on position. Additionally, all players were then separated into either a higher or lower cardiorespiratory fitness group within their position based on the average VO_2peak_ and AT. A one-way Analysis of Variance (ANOVA) was then utilized to examine differences between these groups. Pearson product moment correlations were then used to examine the relationship between physiological measures and performance data as a whole as well as divided by position. Data were evaluated using SPSS Statistics version 22 (SPSS, Armonk, NY, USA). Statistical significance was set at *p* < 0.05. Data are presented as means and standard deviations. Correlations were considered high (0.80–1.00), moderately high (0.60–0.79), moderate (0.40–0.59) or low (0.20–0.39) [18].

### 2.2. Results

Descriptive data for the entire sample is presented in Table 1. For all pitchers, average VO_2peak_ was 48.13 ± 5.30 (mL/kg/Min). For all pitchers VO_2peak_ was found to have a low-moderate relationship (*p* < 0.05) between WHIP (*r* = −0.484, *p* ≤ 0.01), K (*r* = 0.572, *p* ≤ 0.01) BB/9 (*r* = −0.358, *p* ≤ 0.05) and W (*r* = 0.550, *p* ≤ 0.01). A strong relationship (*r* = 0.602, *p* ≤ 0.01) was found between VO_2peak_ and K/BB. Additionally, low, but significant correlations were also found between AT and K (*r* = −0.328, *p* ≤ 0.05), K/BB (*r* = −327, *p* ≤ 0.05) and W (*r* = 0.358, *p* ≤ 0.05).

**Figure 1:**
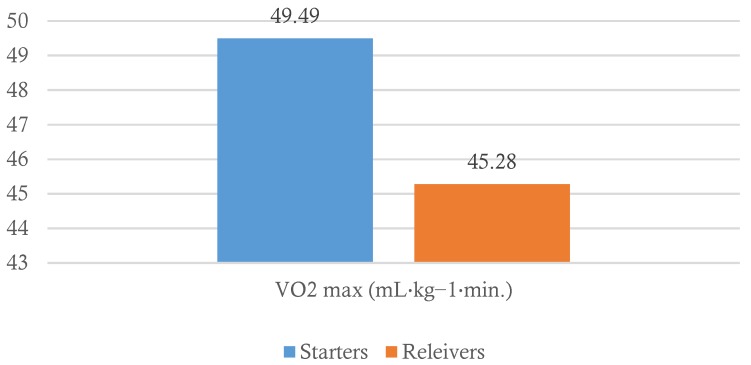
Differences in VO_2 max_ between Starters and Relievers.

When comparing S *vs.* R pitchers there was a significant effect for position, *t* = 2.50, *p* ≤ 0.01, with S pitchers demonstrating higher VO_2 max_ values (49.49 ± 4.59) when compared to relievers (45.28 ± 5.72) as presented in Figure 1. However, our analysis revealed no significant differences in AT between S and R.

**Figure 2:**
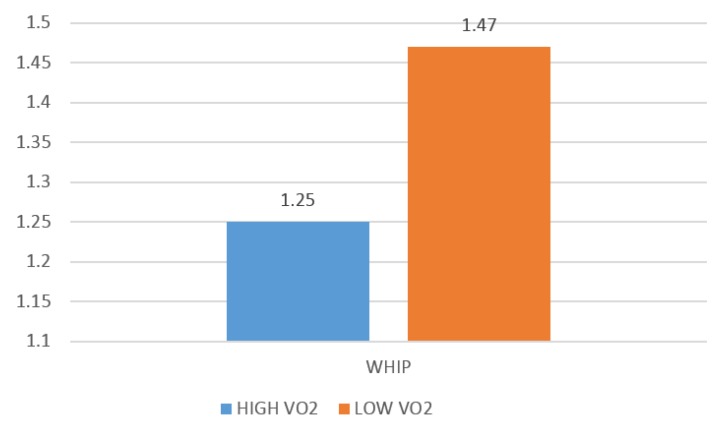
Differences in walks and hits per inning pitched (WHIP) amongst S pitchers with higher and lower VO_2 max_.

**Figure 3:**
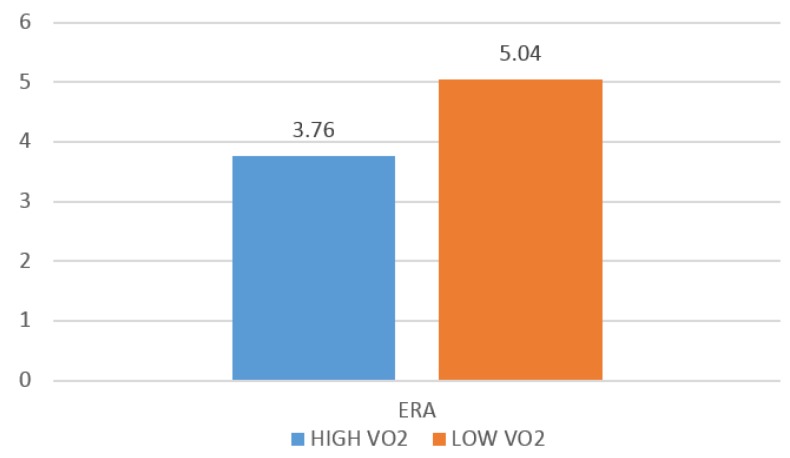
Differences in earned run average (ERA) amongst S pitchers with higher and lower VO_2 max_.

A one-way ANOVA with pairwise comparisons revealed significantly better performance statistics among S pitchers with a VO_2peak_ above the overall group mean in FIP (*F*(3,36) = 3.87, *p* ≤ 0.01, P), WHIP (*F*(3,36) = −4.60, *p* ≤ 0.01), K/BB (*F*(3,36) = 7.25, *p* ≤ 0.01), and ERA (*F*(3,36) = −4.58 *p* ≤ 0.01). Additionally, it was discovered that S pitchers with an AT above the overall group mean demonstrated significantly better performance as measured by WHIP (*F*(3,36) = 2.54, *p* ≤ 0.05), presented in Figure 2, and ERA (*F*(3,36) = 2.52, *p* ≤ 0.05), presented in Figure 3. When comparing R pitchers above and below VO_2peak_ the only significant difference discovered in measures of pitching performance was in HR/9 (*F*(3,36) = 5.06, *p* ≤ 0.05).

Among starting pitchers, Pearson product-moment correlations revealed low- high relationships between VO_2 max_ and BB/9 (*r* = −0.416), HR/9 (*r* = −0.478, *p* ≤ 0.05), W (*r* = 0.548), FIP (*r* = −0.567, *p* ≤ 0.01), K’s (*r* = 0.572, *p* ≤ 0.01), H/9 (*r* = −0.589, *p* ≤ 0.01), K/9 (*r* = 0.614, *p* ≤ 0.01), ERA (*r* = −678, *p* ≤ 0.01), and WHIP (*r* = −0.685, *p* ≤ 0.01). Low correlations were also found between AT and WHIP (*r* = −431, *p* ≤ 0.05) and ERA (*r* = −431, *p* ≤ 0.05). When separately analyzing R pitchers the only significant relationships (*r* = 0.592, *p* ≤ 0.05) was found between FIP and VO_2 max_. This relationship indicated that R pitchers with a higher VO_2 max_ had a higher FIP. No other significant relationships between VO_2 max_ or AT and performance were discovered among R pitchers.

### 2.3. Discussion

The results of this study indicate that ML S pitchers are more aerobically fit than R pitchers and that several pitching performance variables appear to be related to greater VO_2 max_. For S pitchers only, there was a strong, significant correlation between VO_2 max_ and FIP, WHIP, and ERA. There was also a moderate but significant relationship between AT and both WHIP and ERA for S pitchers only. For R pitchers no significant relationships were discovered between either VO_2 max_ and AT for any of the selected measures of used in our analysis. In contrast, the sole correlation among R pitchers in this study was found when this group was subcategorized into high/low VO_2 max_. It was discovered that R pitchers with a higher VO_2 max_ actually pitched worse than those with a lower VO_2 max_ based on FIP. After analysis of R pitchers data it can be concluded that cardiorespiratory fitness may not necessarily have a positive impact on successful pitching performance among R pitchers. While the results may simply be a product of on-field demands, or due to different training and conditioning methods performed between S and R pitchers, it may also suggest that aerobic fitness has a larger impact on pitching performance for the S pitcher at the ML level.

In an attempt to identify key fitness benchmarks related to performance, further evaluations were performed on S pitchers only. The S sample was divided into higher and lower VO_2 max_ groups (greater than or less than 48.13 (mL/kg/min)) to examine differences in performance in FIP, WHIP, and ERA. The higher fitness group (*n* = 15, VO_2_ = 50.19 ± 4.23) had an average WHIP of 1.25 ± 0.18, FIP of 3.61 ± 0.66, and average ERA of 3.77 ± 1.02. The WHIP and ERA results were significantly better (*p* < 0.05) than the lower fitness group (*n* = 12, VO_2_ = 48.84 ± 4.97) which were found to have an average WHIP of 1.48 ± 0.17 and ERA of 5.04 ± 0.97. The higher fitness group also had a better K/9 (*p* < 0.05). In summary, S pitchers exhibiting above average VO_2 max_, pitched considerably better than S pitchers with below average VO_2 max_.

The physiological demands on S pitchers are greater than R pitchers due to the higher workload and duration of time they are expected to perform. This may result in a greater reliance on overall cardiorespiratory fitness and endurance to sustain performance throughout the duration of a game. The apparent lack of importance related to both VO_2 max_ and AT for the R pitcher is not surprising and provides additional support for constructing position or role-specific exercise programs. It makes sense that the relationship between VO_2_ and AT and pitching performance measures may simply indicate the need for higher levels of endurance for S pitchers and not specific requirements for successful pitching performances for R pitchers.

The current findings support the need for personalized in-season conditioning programs dependent on the specific role a pitcher holds on their team. The S pitcher is usually on a 5 day rotation involving four days in between each pitching outing. When the major emphasis of conditioning programs is placed on minimizing reductions in power and strength over the course of the season, the use of slow, long distance runs could be counter-productive. The authors postulate that one high intensity interval training session between each pitching outing may be sufficient to help maintain aerobic fitness and maximize recovery. This approach may also serve to minimize reductions in power and strength and interference during power development and strength training sessions performed during the four days between pitching outings.

Conditioning programs for R pitchers should not mimic S pitchers, even though some R pitchers are expected to pitch multiple innings and sustain higher pitch counts. Practitioners must determine whether or not this type of R pitcher should fall under a modified conditioning program similar to a S pitcher. In this case, the R pitcher would be allowed more days to rest following an outing making it more conducive to higher intensity conditioning sessions. The day following a long outing where a R pitcher has the day off might be best to incorporate more intense conditioning sessions. It should also be noted that more extensive modifications in conditioning programs might be required to meet anthropometric needs/goals.

In summary, this is the first known study that examines aerobic capacity among ML pitchers and its potential impact on pitching performance. When interpreting the current findings it is important to realize that there are many uncontrollable variables that become more evident in field experiments. First, successful pitching performance at the professional level is certainly not identified by one single performance measure. There are many variables that lead to a positive pitching performance, making it difficult to come to precise conclusions on how much of an impact physical fitness really has on performance statistics in this analysis. In addition, pitching mechanics play an important role in successful pitching at any level. Therefore, the evaluation of the statistical findings should consider the complexity of pitching performance in general. Furthermore, it is important to note that subjects exercised to voluntary exhaustion. Subsequently, it is possible that a true VO_2 max_ may not have been reached due to lack of motivation. All of these factors should be considered when evaluating the outcomes of this study.

## 3. Conclusions

### Practical Applications

The current data suggests that metabolic training among ML baseball starting pitchers should focus on improving both aerobic capacity and anaerobic threshold. At the higher levels of play adequate training time should be devoted to maximizing these physiological parameters to improve chances of successful performance. Concurrent training strategies should focus on maximizing both physiological variables while attempting to minimize any potential interference in maximizing power. High intensity interval training seems to be the most efficient mode of training to improve a starting pitcher’s aerobic capacity. Neglecting important physiological components can have a detrimental effect on performance but athletes need evidence-based guidance to ensure productivity and a return on their training efforts.

Strength and conditioning professionals should work closely with pitchers, coaches, and organizational management to design and implement appropriate training strategies for pitchers at various levels. The reliance on research such as the current analysis ensures that training programs are evidence-based and effective. For specific examples of proposed conditioning programs for pitchers, further examination of published works are suggested [7,8,20,21].

## Figures and Tables

**Table 1:** Descriptive data.

Anthropometric and Fitness Variables	All Pitchers (*n* = 40) Mean ± SD	S Pitchers (*n* = 27) Mean ± SD	R Pitchers (*n* = 13) Mean ± SD
Age (YEARS)	28.03 ± 5.17	27.33 ± 5.46	29.46 ± 4.31
Weight (KG)	100.06 ± 6.80	99.27 ± 5.75	101.75 ± 8.59
Height (CM)	191.19 ± 5.11	192.28 ± 5.00	188.92 ± 4.80
Estimated Percent Body fat (%BF)	15.28 ± 3.45	14.21 ± 2.45	17.5 ± 4.2
Lean BODY mass (lBM) (KG)	84.63 ± 4.54	85.07 ± 3.85	83.72 ± 5.79
Estimated Max. Heart Rate (HR)	191.98 ± 5.16	192.66 ± 5.46	190.53 ± 4.31
VO_2 max_ (mL·kg^−1^·min.)	48.13 ± 5.30	49.49 ± 4.59	45.28 ± 5.71
Anaerobic threshold (AT)	37.31 ± 7.87	38.63 ± 7.03	34.57 ± 9.06
HR at VO_2 max_	184.28 ± 9.70	182.33 ± 9.06	188.31 ± 10.09
HR at Anaerobic Threshold	162.72 ± 13.83	161.11 ± 11.97	166.08 ± 17.13
